# CD39 in the pathogenesis of neuro-Behçet disease: is it an immune regulator marker?

**DOI:** 10.3389/fimmu.2026.1779249

**Published:** 2026-03-09

**Authors:** Khadija Bahrini, Amani Stiti, Sana Boughariou, Meriam Belghith

**Affiliations:** 1 Laboratory of Transmission, Control and Immunobiology of Infections, Institut Pasteur de Tunis, Tunis, Tunisia; 2 Department of Anesthesiology and Intensive Care, Military Hospital of Tunis, Tunis, Tunisia

**Keywords:** adenosine, CD39, immune regulation, inflammation, NBD

## Abstract

Neuro-Behçet’s disease (NBD) is a severe neurological manifestation of Behçet’s disease (BD), a multisystemic inflammatory vasculitis affecting multiple organs. NBD presents complex immunopathological features involving both innate and adaptive immune responses. CD39 (ectonucleoside triphosphate diphosphohydrolase-1, ENTPD1), an ectoenzyme expressed on various immune cells, has emerged as a critical regulator of inflammation through its role in hydrolyzing extracellular ATP to immunosuppressive adenosine. This mini-review explores the role of CD39 in modulating neuroinflammation in NBD, focusing on its function in immune cell subsets, its dual role in inflammation and regulation, and its potential as a therapeutic target. It synthesizes the role of CD39 in neuroinflammatory disorders, with a specific focus on Neuro-Behçet’s Disease (NBD). We examine the dual role of CD39 expression in driving inflammation and immune regulation, and highlighting its potential as a therapeutic target.

## 1 Introduction

Behçet’s disease (BD) is a multisystem vasculitis that presents with variable clinical manifestations across different disease episodes. The hallmark features include oral aphthous ulcers, genital ulcers, and ocular lesions, though the condition can affect multiple organ systems including vascular structures (both arteries and veins), joints, the gastrointestinal tract, and the nervous system (1). The neurological form of this disease named Neuro-Behçet (NBD) represents the most severe and potentially fatal manifestation of BD. Though it occurs in only a minority of patients, with prevalence rates ranging from 3% to 30% of all BD cases (2). NBD can be categorized into two distinct clinical subsets based on anatomical involvement: the Parenchymal NBD (p-NBD) and the Non-parenchymal NBD (Non-p-NBD) (3).

The p-NBD form is characterized by magnetic resonance imaging (MRI) abnormalities that directly involve brain parenchyma, including the brain stem, spinal cord, and cerebral hemispheres. The Non-p-NBD presents with vascular complications such as intracranial aneurysms and cerebral venous thrombosis. These manifestations are typically considered an extension of the broader cerebrovascular involvement that can occur in BD (4).

Based on the clinical progression patterns of the disease (5, 6), NBD patients can be categorized into two types: Acute NBD, which presents with sudden onset and intense symptoms, and Chronic progressive NBD, which exhibits a slow, steady worsening with continuous neurological decline over an extended period (4). Moreover, Neuro-Behçet’s disease represents a major clinical challenge due to its unpredictable course, high morbidity, and potential for irreversible neurological damage. In routine clinical practice, NBD often presents with heterogeneous neurological manifestations that overlap with other inflammatory and vascular disorders of the central nervous system, frequently leading to diagnostic delays.

To this point, established clinical protocols for NBD diagnosis have not been validated, and the immunopathological mechanisms of NBD are still not fully characterized. Treatment decisions are largely empirical and based on extrapolation from systemic Behçet disease or other neuroinflammatory conditions, underscoring the need for improved immunopathological understanding that could inform clinical stratification and management. In addition, laboratory findings show a disrupted central nervous system with inflammation and tissue damage, indicated by cerebrospinal fluid (CSF) abnormalities like a high cell count (pleocytosis), increased total protein, and elevated IL-6 concentrations (7).

Previous studies have highlighted the purinergic signaling pathway as a crucial modulator of immune responses in inflammation. CD39 plays a pivotal role in this pathway by hydrolyzing pro-inflammatory ATP and ADP to AMP, which is further converted into adenosine by CD73. This cascade results in suppression of immune activation and promotion of immune tolerance (8). Given the strong inflammatory environment observed in NBD, understanding the function of CD39 in this context offers insights into novel mechanisms and therapeutic strategies. In this mini review, we summarize existing evidence related to the CD39 pattern in NBD.

## 2 Purinergic signaling and CD39 function

Purinergic signaling constitutes a fundamental, evolutionarily ancient pathway that governs cellular communication, playing especially critical roles in immune system regulation (9). ATP functions as a key purine signaling molecule that is normally contained within cellular compartments during healthy conditions (10). Cellular stress or injury triggers ATP release into the surrounding extracellular environment, initiating inflammatory responses (11). A series of enzymes, predominantly CD39, breaks down this extracellular ATP to ADP and AMP which is subsequently converted to adenosine by CD73. CD39 has been identified as an emerging “immune checkpoint modulator” that can suppress both antitumor and anti-inflammatory immune activities (12). Clinical investigations have revealed that CD39 has a critical involvement across multiple disease processes, establishing its significant potential as a novel therapeutic target (13).

CD39, expressed on immune cells such as T cells, B cells, dendritic cells, macrophages, and NK cells, degrades extracellular ATP and ADP into AMP. CD73 then converts AMP into adenosine, a potent immunosuppressive molecule that binds to P1 receptors (A1, A2A, A2B,and A3). Among these, the A2A receptor plays a central role in dampening T cell activation and cytokine production (8).

The ATP adenosine axis serves as a metabolic switch that governs immune cell fate and function, shifting the balance from inflammation to regulation (14). In the context of central nervous system (CNS) inflammation, such as NBD, this mechanism may be crucial in controlling excessive immune responses that contribute to neural damage.

## 3 CD39 and regulatory T cells in inflammatory disorders

**Figure 1:**
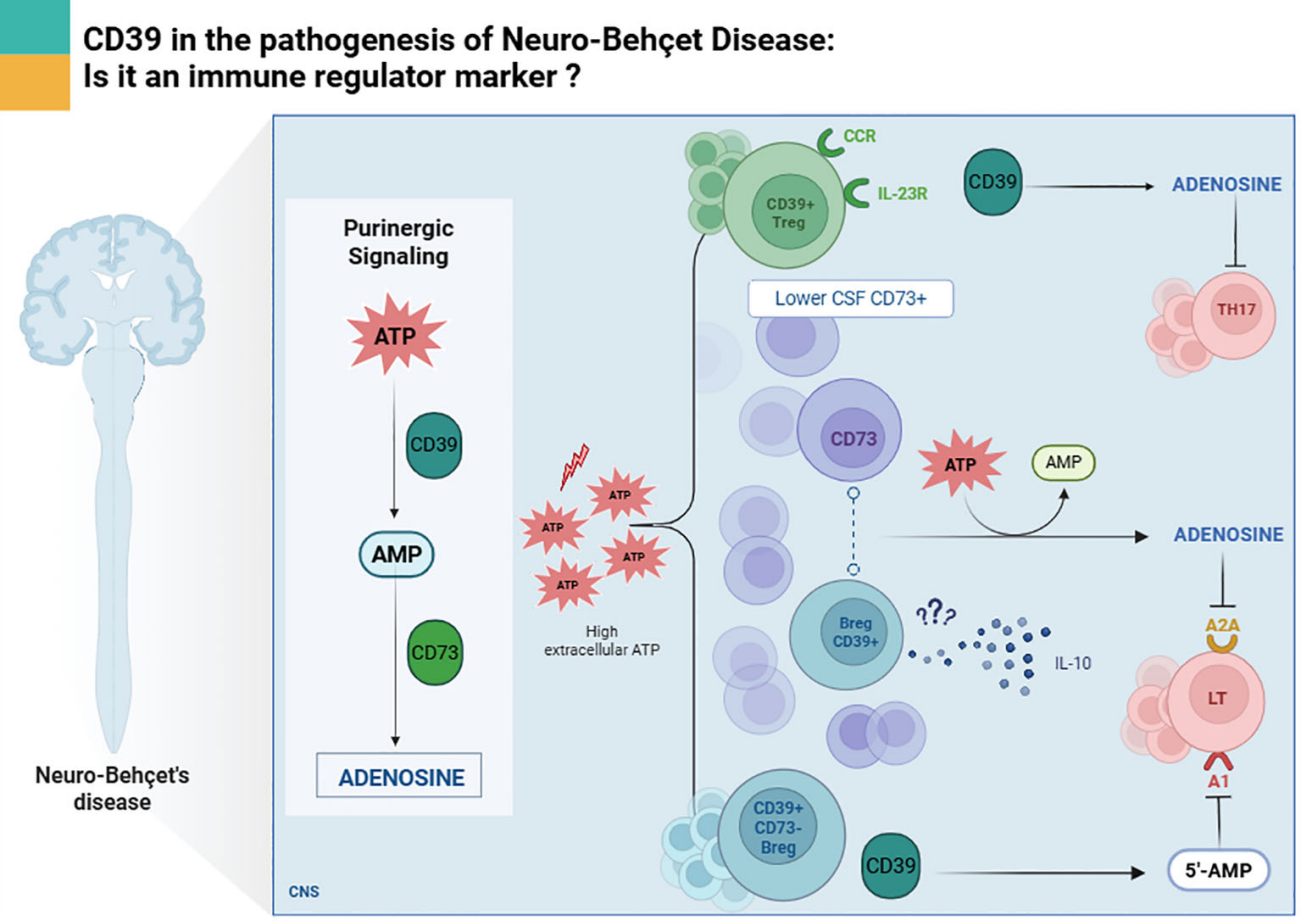
CD39-mediated regulatory pathways in Treg and Breg cells in the central nervous system during Neuro-Behçet disease.

Regulatory T cells (Tregs) are essential for maintaining immune homeostasis and preventing autoimmunity. CD39 is preferentially expressed on a subset of Tregs (CD4+CD25+Foxp3+CD39+), which possess superior suppressive capacity compared to CD39- Tregs (15). Although research examining CD39 involvement in NBD is scarce, CD39+ Tregs appear to be particularly important in NBD, given the predominance of Th1 and Th17 immune responses. Our prior work revealed elevated CD39 mRNA expression in the cerebrospinal fluid (CSF) of NBD patients compared to healthy controls (16). These cells can suppress IL-17 production, a key driver of inflammation in NBD (**Figure 1**), through adenosine-mediated mechanisms (17). Moreover, CD39+ regulatory T cells express receptors such as CCR6 and IL-23R, which facilitate their migration to inflamed tissues and allow them to limit Th17 persistence by competing for IL-23, an essential cytokine for Th17 maintenance while secreting IL-10 (18).

Moreover, CD39 expression stabilizes Treg function in inflammatory environments. Studies have shown that CD39+ Tregs maintain Foxp3 expression and suppressive capacity even under inflammatory conditions, whereas CD39- Tregs are prone to lose their regulatory phenotype (19). This suggests that CD39 not only distinguishes highly suppressive Tregs but also potentially supports their functional viability in pathological conditions such as NBD.

## 4 CD39 and regulatory B cells in neuroinflammation

While B cells are traditionally associated with antibody production and T cell activation, a subset of B cells known as regulatory B cells (Bregs) can modulate immune responses through cytokine secretion and cell-cell interactions (20). CD39 expression has been identified on Bregs (CD19+CD25+CD39+), enhancing their ability to suppress effector T cell responses. When B cells become activated in an environment containing extracellular ATP, they increase their expression of CD39 while simultaneously decreasing CD73 levels, resulting in predominant production of 5′-AMP with minimal adenosine generation (21). Despite this altered enzymatic profile, these B cells effectively suppress T-cell proliferation and cytokine secretion through a mechanism likely mediated by 5′-AMP signaling. Given that 5′-AMP has been identified as an agonist for the A1 receptor (A1R) (22), it has been reported that A1R-expressing T cells experience inhibition through two distinct pathways: adenosine acting on A2A receptors and B cell-generated 5′-AMP acting on A1 receptors (23). An inflammatory process depending on IL-6, IL1-β and GM-CSF has been previously shown to promote B cell differentiation into B reg phenotype (24). We previously reported that CD39 ectoenzyme is preferentially expressed on B cells. This expression is more abundant in NBD CSF as compared to multiple sclerosis patients (16). This potentially argues in favor of a role of B reg cells, since it has been shown that an inflammatory environment depending on pro-inflammatory cytokine such as IL-6 and IL-1β could promote this B subpopulation (23, 24).

Based on this result we surmise that Bregs CD39+ may contribute to immune regulation by producing IL-10 and promoting Treg expansion in NBD especially that IL-10 is shown to be higher in the CSF of NBD patients as compared to Multiple Sclerosis patients (25). CD39 facilitates this function by degrading ATP and reducing pro-inflammatory signaling. Although CD73 expression appears to be weak in NBD (16), the conversion of AMP to adenosine may still occur through paracrine mechanisms mediated by CD73 expressed on neighboring or target cells (**Figure 1**) (26).

Activated B cells expressing CD39 have been shown to inhibit T cell proliferation *in vitro*, highlighting their potential role in controlling CNS inflammation (23). These findings underscore the multifaceted regulatory functions of CD39 across immune cell types and its relevance in diseases characterized by uncontrolled inflammation, such as NBD.

As summarized in **Table 1**, CD39 expression varies across different immune cell types in NBD, highlighting its context-dependent role in neuroinflammation.

**Table 1:** CD39 expression across immune cells in NBD.

Immune cell	CD39 expression	Functional implication
Tregs (CD4+CD25+Foxp3+)	High in CSF	Suppress Th17
Bregs (CD19+)	High in CSF	Produce IL-10, promote Treg expansion
Th17/Effector T cells	Variable	Context-dependent, can be pathogenic
MS comparison	Reduced CD73	Highlights disease-specific purinergic regulation

Further investigations of CD39 expression on regulatory B cells in both peripheral blood mononuclear cells and cerebrospinal fluid are necessary to elucidate the functional role of this cell population in NBD patients.

## 5 CD39 expression on effector T cells: a double-edged sword

Interestingly, CD39 is not restricted to regulatory T and B cells. Effector T cells, including Th17 cells, may also express CD39, particularly in inflammatory settings. These CD4+CD39+Foxp3- cells retain ATP-hydrolyzing capacity but lack immunosuppressive function. In this context, Liberal and collaborators have shown that Th17 cells expressing CD39 are significantly reduced and unable to produce AMP or adenosine, consequently impairing their ability to regulate target cell expansion and IL-17 secretion in juvenile autoimmune liver disease (27). In autoimmune disorders like Crohn disease and rheumatoid arthritis, these cells are associated with disease activity (28, 29). Moreover, CD4+CD39+CD161+ T cells have been identified as Th17 precursors. The co-expression of CD39 and CD161 initiates sphingomyelinase activity, leading to increased intracellular ceramide, which activates STAT3 and mTOR pathways, driving Th17 differentiation and IL-17 production (29). This dual role of CD39 highlights its context-dependent function acting as a brake in regulatory cells and a potential amplifier in certain effector subsets.

While considerable research has evaluated CD39 expression on effector T cells in peripheral blood mononuclear cells from autoimmune disease patients, no studies to date have, to the best of our knowledge, examined this ectoenzyme expression on Th17 cells in the CNS or CSF of individuals with NBD. Further investigation is necessary to clarify the role of this ectoenzyme in this neuroinflammatory disorder. The dynamic nature of CD39 function implies that in NBD, when expressed on effector cells, it may drive ongoing inflammation, while its expression on regulatory cells might serve as a mechanism for restoring immune homeostasis.

## 6 CD39 in other CNS inflammatory diseases: multiple sclerosis

Given the clinical similarities between NBD and MS during early disease stages, we previously conducted a comparative analysis of CD39 expression patterns in both conditions to help contextualize potential disease-specific immune profiles rather than infer shared mechanisms. Our findings in peripheral blood mononuclear cells revealed significantly lower CD39 expression in relapsing-remitting MS patients compared to those with NBD. Additionally, CD73 expression was similarly reduced in the RRMS cohort as compared to NBD (16). We can hypothesize that these cells have regulatory functions and reduced in PBMC of MS patients while the diminished CD39+ T cell population in RRMS was restored following fingolimod therapy, which enhanced CD39+Foxp3+ regulatory T cell numbers in the peripheral blood of MS patients (30). This observation underscores the impact of immunomodulatory treatments on purinergic pathways in MS and suggests that therapeutic context should be considered when interpreting CD39 expression levels. Similarly, the expansion of CD39+ Tregs following immunomodulatory treatment correlates with reduced inflammation. Previous studies have demonstrated that elevated CD39 levels can inhibit MS progression by suppressing IL-17 secretion through Foxp3+ cells with regulatory characteristics (17). However, these findings should not be interpreted as evidence of identical regulatory mechanisms operating in NBD, since the role and functional consequences of CD39^+^CD73^+^ regulatory T cells in the peripheral blood of NBD patients have not yet been clearly defined. As a result, similar expression patterns may not translate into comparable immunoregulatory functions.

Our previous finding in CSF of MS and NBD patients showed elevated CD39 mRNA expression in both RRMS and NBD patients compared to non-inflammatory neurological disease controls, who exhibited minimal expression of this marker. Despite this shared increase, distinct patterns of immune compartmentalization were observed, as RRMS patients displayed higher CD73 expression in the CSF than NBD patients. Consistent with these molecular findings, flow cytometric analysis confirmed a significant increase in CD39+CD73+ double-positive cells in the CSF of RRMS patients relative to NBD patients (16). Taken together, these results highlight differences in purinergic regulation between MS and NBD that likely reflect disease-specific immune environments rather than direct mechanistic equivalence.

## 7 Therapeutic proposition of targeting CD39 in NBD

Although numerous clinical trials are currently evaluating CD39 and CD73 targeted strategies in cancer (31, 32) and other inflammatory disorders (13). The therapeutic relevance of this ectoenzyme system in NBD remains insufficiently defined. In oncology, CD39 plays a pivotal role in several key processes driving cancer progression, including angiogenesis, metabolic reprogramming, and intercellular communication within the tumor microenvironment (33). Pharmacological inhibition of CD39 leads to the accumulation of extracellular ATP released from stressed or dying cells, thereby amplifying immune-stimulatory signaling cascades (34, 35). Concurrently, reduced conversion of ATP to adenosine limits adenosine-mediated immunosuppression, which may restore or enhance antitumor immune responses (36, 37). In cancer models, CD39-blocking antibodies have been shown to enhance CD4^+^ and CD8^+^ T-cell proliferation and to increase CD8^+^ T-cell and NK cell mediated cytotoxicity against tumor cell lines, highlighting their antitumor potential (38). Nevertheless, despite these promising effects, the clinical translation of CD39 inhibition remains challenging, and its long-term efficacy and safety require further investigation, particularly with respect to potential off-target effects and dysregulated inflammation (15, 39).

In contrast to their role in cancer where CD39 inhibition is used to stimulate immune activation, the applications in autoimmune and inflammatory diseases are more nuanced. In fact, function presents a paradox in autoimmune and inflammatory disease. Through its expression on regulatory T cells and myeloid populations, CD39 produces adenosine that dampens excessive immune activity, thereby helping preserve self-tolerance (40). Yet, this same adenosine-generating capacity can prove detrimental when adenosine accumulates excessively, potentially driving tissue fibrosis and perpetuating chronic inflammatory processes that worsen certain autoimmune pathologies. The therapeutic application of CD39 inhibitors in autoimmune and inflammatory diseases is currently an active area of investigation. The rationale for using these inhibitors is based on their ability to alter purinergic signaling and thereby influence key immunological processes, including immune cell migration, antigen-presenting cell function, and cytokine production by modifying extracellular nucleotide gradients and downstream adenosine generation, which can recalibrate inflammatory responses in auto-immune or inflammatory diseases.

Emerging data show altered expression of CD39 in the CSF of NBD patients, yet the precise distribution of CD39/CD73 across immune cell subset including B cells, T cells along with their functional impact on purinergic signaling in the CNS, is still poorly understood. Importantly, unlike oncology where CD39 inhibition aims to enhance immune activation, therapeutic approaches in inflammatory diseases including NBD are more likely to require selective modulation of the CD39/CD73 adenosine axis rather than blanket inhibition.

Therefore, before rational therapeutic intervention can be designed, it is essential to characterize the phenotype and immunological function of CD39^+^ cellular populations in the CSF and blood of NBD patients, as well as to map how these pathways influence ATP-driven inflammation versus adenosine-mediated immunoregulation. Once these mechanistic foundations are clarified, several therapeutic strategies could be envisaged: First, adenosine receptor agonists to reinforce downstream anti-inflammatory signaling when endogenous adenosine production is insufficient. Second, recombinant CD73 or approaches enhancing CD73 activity to promote the conversion of pro-inflammatory extracellular ATP/AMP into immunoregulatory adenosine. Third, broader modulation of the purinergic axis to dampen ATP-mediated activation of neutrophils and other effector cells that drive NBD pathology. Establishing the cellular landscape of the CD39/CD73 pathway in NBD is therefore a prerequisite for identifying which of these strategies holds true therapeutic potential and for designing targeted interventions tailored to the unique neuro-inflammatory environment of NBD.

## 8 Discussion

NBD represents one of the most severe inflammatory manifestations of Behçet’s disease, yet its immunopathological mechanisms remain incompletely defined (5, 6). The clinical significance of immune dysregulation in NBD lies in its potential to determine the balance between uncontrolled neuroinflammation and compensatory immune regulation within the CNS. Alterations in purinergic signaling may therefore help explain disease severity, persistence of inflammation, and progression in NBD.

This mini-review highlights the emerging relevance of the purinergic signaling pathway, particularly the ectoenzyme CD39, as a central regulator of immune responses within the neuroinflammatory environment of NBD. By integrating evidence from regulatory and effector immune compartments, our synthesis positions CD39 as a critical, context-dependent modulator at the intersection of inflammation and immune regulation in the CNS (8, 9).

Accumulating data indicate that CD39 expression on regulatory T and B cells may represent a compensatory mechanism aimed at restraining excessive ATP-driven inflammation in NBD. The preferential enrichment of CD39^+^ immune cells in the CSF of NBD patients on B reg cells, together with elevated anti-inflammatory mediators such as IL-10, supports the notion of an active but potentially insufficient immunoregulatory response (16, 25). In this regard, CD39^+^ Tregs and Bregs may contribute to limiting Th1/Th17-mediated pathology through ATP hydrolysis and adenosine or AMP dependent signaling (17, 21, 23). However, the persistence of severe neurological damage in NBD suggests that this regulatory axis may be functionally overwhelmed or spatially dysregulated within inflamed CNS compartments.

Importantly, CD39 also emerges as a double-edged molecule when expressed on effector T cell subsets. In inflammatory settings, CD39 expression on Th17 or Th17-precursor cells may promote pathogenic differentiation and sustain IL-17-driven neuroinflammation through non-canonical signaling pathways involving STAT3 and mTOR activation (27, 29). This functional dichotomy underscores the complexity of targeting CD39 therapeutically and highlights the necessity of precisely defining its cell-specific expression and enzymatic activity in NBD.

Comparative insights from MS further underscore the disease-specific nature of purinergic regulation in neuroinflammatory disorders. Accumulating evidence indicates that the cellular distribution of CD39 and the balance between extracellular ATP-degrading and adenosine generating pathways differ substantially between MS and NBD, suggesting fundamentally distinct immunoregulatory landscapes (16). In MS, reduced CD39 expression on peripheral immune cell subsets, particularly regulatory T cells, has been associated with enhanced pro-inflammatory activity and loss of immune tolerance. Notably, several studies have shown that effective immunomodulatory therapies can partially restore the frequency and function of CD39^+^ regulatory T cells, a change that correlates with reduced inflammatory activity and clinical improvement (17, 30).

In contrast, NBD appears to involve a distinct pattern of purinergic dysregulation, potentially shaped by differences in disease pathogenesis, innate and adaptive immune crosstalk, and specific immune activation within the CNS. These differences may influence how extracellular nucleotide signaling contributes to inflammation, immune suppression, and tissue damage in each condition. Collectively, these contrasts highlight that, while purinergic pathways represent a common regulatory axis in neuroinflammation, their functional impact, therapeutic relevance, and clinical implications are highly context-dependent. This may partly explain the divergent disease trajectories and variable therapeutic responses observed between MS and NBD, reinforcing the need for disease-specific interpretation and targeted modulation of CD39-mediated pathways.

Despite growing interest in CD39/CD73-targeted interventions in cancer and inflammatory disorders (12, 13, 31, 32), their relevance in NBD remains largely unexplored. From a clinical perspective, the inflammatory damage observed in NBD is likely driven by sustained immune activation within the CNS. Elevated cytokines such as IL-6 in the CSF reflect this ongoing inflammation and may influence the unbalance between effector and regulatory immune responses. Understanding how CD39 expressing immune cells respond to this environment may help explain why some patients develop progressive neurological deterioration despite immunosuppressive therapy, emphasizing the clinical relevance of dissecting immune regulatory pathways in NBD.

A major limitation in the field is the paucity of functional studies dissecting purinergic signaling at the cellular level within the CNS of NBD patients. Future investigations should prioritize multiparametric analyses integrating phenotypic, transcriptional, and functional approaches in both blood and CSF samples to delineate how CD39 shapes immune cell fate and neuroinflammatory outcomes.

In conclusion, CD39 emerges as a critical immune regulator in NBD, with the potential to influence both pathogenic and protective immune responses. Its role in hydrolyzing ATP and generating adenosine positions it at the interface of inflammation and immune regulation. The expression of CD39 on Tregs, Bregs, and effector T cells determines its net impact on disease progression.

Understanding the nuanced role of CD39 in NBD not only sheds light on disease mechanisms but also opens new perspectives for targeted therapy. Future studies should focus on characterizing CD39+ cell subsets in NB patients, elucidating their functional roles, and exploring therapeutic modulation of the CD39-adenosine axis.
